# Patient safety in orthodontic care: a scoping literature review with proposal for terminology and future research agenda

**DOI:** 10.1186/s12903-024-04375-7

**Published:** 2024-06-18

**Authors:** Nikolaos Ferlias, Ambrosina Michelotti, Peter Stoustrup

**Affiliations:** 1 Section of Orthodontics, Department of Dentistry and Oral Health, Aarhus University, Aarhus, Denmark; 2 Department of Neurosciences, Reproductive Sciences and Oral Sciences, Section of Orthodontics and Temporomandibular Disorders, University of Naples Federico II, Naples, Italy; 3 Private Practice, Brighton, UK

**Keywords:** Systematic review, Patient safety, Orthodontics, Patient safety incidents, Patient harm, Adverse events

## Abstract

**Background:**

Knowledge about patient safety in orthodontics is scarce. Lack of standardisation and a common terminology hinders research and limits our understanding of the discipline. This study aims to 1) summarise current knowledge about patient safety incidents (PSI) in orthodontic care by conducting a systematic literature search, 2) propose a new standardisation of PSI terminology and 3) propose a future research agenda on patient safety in the field of orthodontics.

**Methods:**

A systematic literature search was performed in the main online sources of PubMed, Web of Science, Scopus and OpenGrey from their inception to 1 July 2023. Inclusion criteria were based on the World Health Organization´s (WHO) research cycle on patient safety. Studies providing information about the cycle’s steps related to orthodontics were included. Study selection and data extraction were performed by two of the authors.

**Results:**

A total of 3,923 articles were retrieved. After review of titles and abstracts, 41 articles were selected for full-text review and 25 articles were eligible for inclusion. Seven provided information on the WHO’s research cycle step 1 (“measuring harm”), twenty-one on “understanding causes” (step 2) and twelve on “identifying solutions” (step 3). No study provided information on Steps 4 and 5 (“evaluating impact” or “translating evidence into safer care”).

**Conclusion:**

Current evidence on patient safety in orthodontics is scarce due to a lack of standardised reporting and probably also under-reporting of PSIs. Current literature on orthodontic patient safety deals primarily with “measuring harms” and “understanding causes of patient safety”, whereas less attention has been devoted to initiatives “identifying solutions”, “evaluating impact” and “translating evidence into safer care”. The present project holds a proposal for a new categorisation, terminology and future research agenda that may serve as a framework to support future research and clinical initiatives to improve patient safety in orthodontic care.

**Registration:**

PROSPERO (CRD42022371982).

**Supplementary Information:**

The online version contains supplementary material available at 10.1186/s12903-024-04375-7.

## Introduction

For decades, patient safety has been recognised as a healthcare discipline. However, the awareness-raising publication of “To Err Is Human” by the Institute of Medicine Committee on Quality of Health Care in the US drew considerable attention to this important aspect of healthcare [1, 2]. In this publication, experts estimated that in the US in any given year as many as 98,000 people die from medical errors that occur in hospitals [1]. The definition of patient safety by the World Health Organization (WHO) from 2009 is: “the freedom for a patient from unnecessary harm or potential harm related to healthcare” [2]. Similarly, in their report, Kohn et al. recognised safety as “freedom from accidental injury” [1]. In this context, a patient safety incident (PSI) is an event or circumstance that could have resulted or did result in unnecessary harm to a patient [2].

Patient safety is a crucial aspect of healthcare that seeks to minimise preventable harm, accidents, complications and adverse events (AEs). AEs are defined as injuries resulting from poor management practices that could have been prevented but are not attributed to an underlying disease process [2, 3]. The WHO classifies certain AEs as "never events", which are serious incidents that should not occur given the presence of strong systemic safety measures [4]. Never events can have a profound impact on patients, and their prevention is a key objective of healthcare organisations. In this context, patient safety aims to limit the impact of AEs adverse events and promote the avoidance of preventable harm.

Patient safety is a priority from the patient’s perspective, and for care providers it falls in line with the Hippocratic Oath ("primum non nocere"), which is an important element of modern healthcare. Patient safety initiatives analyse characteristics and features of healthcare systems that may lead to the occurrence of AEs. These features are latent risks that may be of any nature from a soft tissue laceration or a loose wire to inhalation of an orthodontic appliance [5]. Throughout most healthcare treatment courses, multiple latent risks exist and this makes patient safety multifactorial and complex. When an AE occurs, patient safety does not aim to punish but rather to investigate how and why the protective barriers failed [6, 7].

Improving the quality of care is a road that passes through patient safety. Additionally, patient safety has additional psychosocial and financial benefits. Dealing with the consequences of an adverse event has an economic cost to the practitioner, the patient and society. By improving patient safety, dental practitioners increase their quality of care, which is associated with safer and better treatment outcomes [8–10]. In addition, it affords increased legal security by minimising the risk of legal claims [6].

Knowledge about patient safety in dental care and orthodontics in particular is scarce. The absence of patient safety guidelines in orthodontics is a major concern. This issue is further complicated by the absence of standardized terminology in the field, challenging the development of consistent safety protocols. Additionally, there is a noticeable lack of research and publications in this area, which hinders progress in developing effective, evidence-based strategies to ensure patient safety in orthodontic care [11]. Therefore, an urgent need exists for studies in the field of orthodontics in particular [2, 3, 12]. Among others, the lack of a common language among orthodontic caregivers ultimately hinders research and limits our understanding of the discipline [13, 14]. The aims of this study were to 1) summarise current knowledge about PSIs in orthodontic care by performing a systematic literature search; 2) propose a new standardisation of PSI terminology; 3) propose a research agenda on patient safety in the field of orthodontics that may serve to further develop and provide direction for future research on the subject.

## Materials and methods

### Protocol and registration

Prior to the initiation of the project, the study protocol was registered with PROSPERO (reg. no. CRD42022371982). No ethical approval was deemed necessary.

### Search strategy

A systematic literature search was performed in the main online sources of MEDLINE (through PubMed), Web of Science, Scopus as well as the System for Information on Grey Literature in Europe (Open-Grey) from their inception to 1 July 2023. No language limitation was set in the search, and all types of eligible human studies were included.

**Fig. 1:**
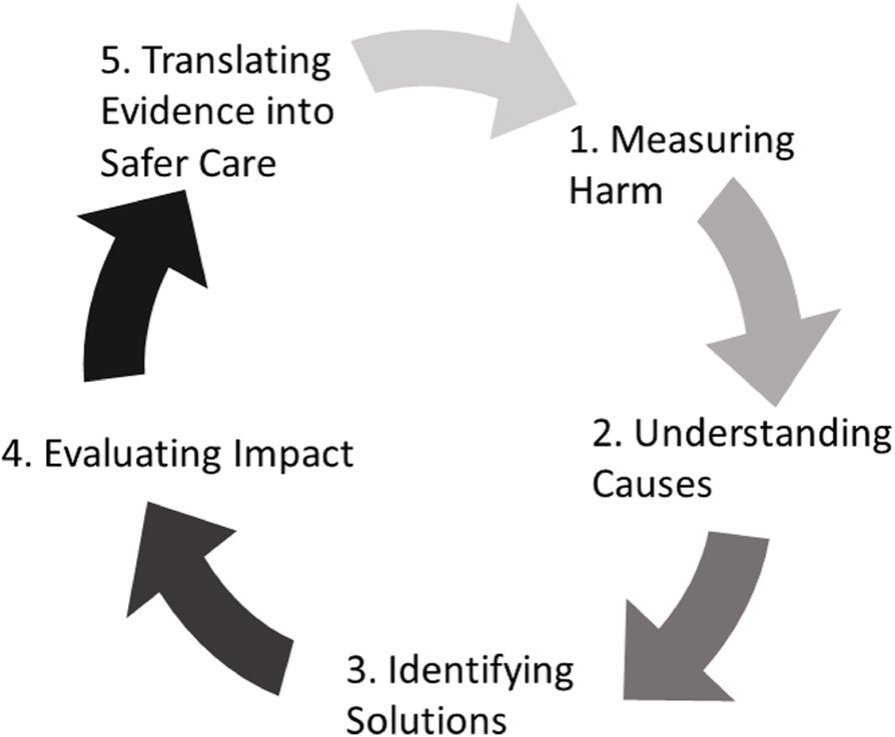
The World Health Organization’s research cycle on patient safety consisting of five steps with the main goal of measuring harm and its causes while identifying solutions and their impact. Ultimately, this evidence should lead to safer care with a set of actions and preventable measures

The inclusion criteria for articles were based on the WHO research cycle on patient safety [15, 16]. The various steps of the cycle aim to measure harm and identify causes while identifying solutions to improve patient safety. The ultimate goal is to translate evidence into safer care (Fig. 1). Only studies that provided relevant information in at least one of the following categories were eligible for inclusion in this systematic review:

1. Measuring harm: Studies characterising and/or reporting on the occurrence of AEs or orthodontic-related patient harm.
2. Understanding causes: Reports focusing on understanding causes leading to patient harm and AEs from orthodontic care.
3. Identifying solutions: Studies identifying solutions that are effective in reducing the occurrence of AEs and patient harm.
4. Evaluating impact: Studies evaluating the effectiveness of solutions in terms of impact, affordability and acceptability.

Only full-text articles were included. In addition, studies dealing with patient safety from a general dental-care perspective were included only if they were directly relevant to orthodontic care and the WHO research cycle. For example, although studies on oral surgery were excluded, wrong-tooth-extraction studies or articles investigating the light-curing safety on patients were included owing to their relevance to orthodontics.

The following MESH terms were used for the systematic search:

(((orthodontic*) OR (dental)) AND (patient safety)) AND ((((((((((((((((((((((((((harm) OR (risk*)) OR (malpractice)) OR (adverse event*)) OR (adverse effect*)) OR (never event*)) OR (iatrogenic)) OR (damage)) OR (incident*)) OR (accident*)) OR (delay* diagnos*)) OR (misdiagnosis)) OR (complication*)) OR (allerg*)) OR (infection)) OR (failure)) OR (error*)) OR (white spot lesion*)) OR (root resorption)) OR (relapse)) OR (decalcification)) OR (caries)) OR (periodontal disease)) OR (nerve damage)) OR (injury)) OR (temporomandibular joint dysfunction)).

### Data extraction

After removal of duplicates, all results returned from the systematic literature search were initially screened by their title to establish their relevance. The second filtering decided relevance for inclusion based on the content of the abstract. Finally, the third filtering level was applied to the main text, and the remaining studies were then included in the review. All screening was performed independently by one of the authors (NF) and was later re-checked by another author (PS). Any disputes in study selection were addressed and resolved through discussion between the reviewing authors. On all included studies the main outcome/result was recorded. This was studies investigating prevalence (“measuring harm”- step 1) or assessing contributing factors (“understanding causes”-step 2). For all studies providing information on the cycle’s step 3 (“identifying solutions”), all recommended solutions to prevent harm were also noted. Due to the nature of the data in the included studies, no risk of bias assessment was possible. For the same reason, no quantitative synthesis and meta-analysis was performed. Based on these findings, the intention to conduct a systematic review was revised to a scoping literature review instead [17].

## Results

### Study selection

**Fig. 2:**
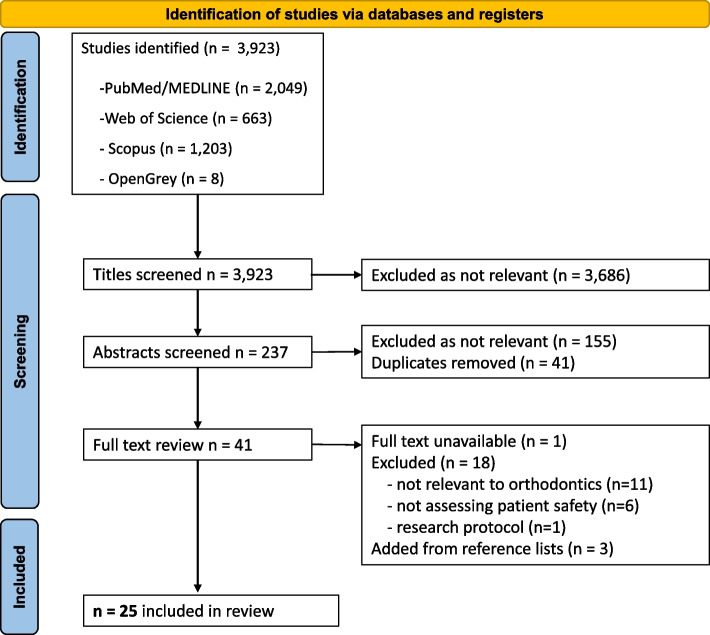
PRISMA flowchart diagram of the systematic literature search and inclusion procedure

A total of 3,923 studies were identified from the systematic search and imported into Excel (Microsoft®, USA) (PubMed n = 2,049, Web of Science n = 663, Scopus n = 1203 and OpenGrey n = 8). Among the 3,923 articles, 237 were deemed relevant according to the inclusion criteria after screening their titles. Filtering by abstracts, left 41 articles for inclusion after removal of the duplicates. In one case, the full-text of an article was unavailable and it was therefore excluded [18]. Three relevant articles found in the reference lists were also added [4, 14, 19]. Finally, 25 studies were included as they were found to provide information within any of the categories of the WHO’s research cycle on patient safety related to the orthodontic field (flowchart presented in Fig. 2).

### Study characteristics

Study characteristics are shown in Table 1. Nine of the included papers were retrospective studies of AEs studying: eye wear protection and ocular trauma in orthodontic practice [19], clinical evaluation of a locking orthodontic facebow [20], adverse reactions to dental materials [3], case reports of latex allergy [21], wrong tooth extraction claims [4], dental and orthodontic PSIs in a UK register [7] and a Finnish register [8], adverse reactions to dental devices reported at the US Food and Drug Administration [9] and investigation of monomer release from orthodontic adhesives [22].

**Table 1:** Included studies with main information and study characteristics (design, country), main outcomes and information provided based on the WHO’s research cycle on patient safety

		Study	Study Information (type, country)	Results/Outcome	WHO's cycle	Solutions (WHO's Cat 3)
retrospective review of AEs	1	Sims et al. 1993	Postal survey on eye-protection use and incidence of ocular trauma in orthodontic practice, UK	Ocular injuries were reported in 37.7% of respondents involving orthodontists, assistants and patients	1, 2, 3	Patients as well as clinicians and their assistants need to wear eye protection goggles which should comply with the British Standards 2092. Especially, during debond or acrylic adjustments using rotary instruments
	2	Samuels et al.2000	Clinical evaluation of a locking orthodontic facebow, UK and Switzerland	The locking facebow was easy to fit and wear by all patients and resulted in less than 1% detachment during night	2,3	It is recommended that a self-releasing extra oral traction system as well as a locking orthodontic facebow should be the system of choice for all extra oral traction patients
	3	Scott et al. 2004	National survey of adverse reactions to dental materials using the Dental Adverse Reaction Reporting Form, UK	Base metals account for 24.6% of all patient adverse reactions to metal (of which 9.7% is Nickel)	2	-
	4	Raggio et al. 2010	Two case reports of latex allergy in paediatric patients, Brasil	When appropriate measures are taken during treatment of latex-allergic patients contact dermatitis and anaphylactic shock can be prevented	2, 3	Dental professionals should avoid the use of any materials containing natural rubber latex. They could also consider the desensitization method to safely expose allergic patients to the irritants gradually
	5	Peleg et al. 2011	Root cause analysis of wrong tooth extraction in 54 insurance claims, Israel	In two thirds of all claims there was an identification error leading to wrong tooth extraction. Provides possible solutions that increase safety	1, 2, 3	To include brief description of tooth to be extracted, confirm with the patient and verify tooth, confirm tooth position before and after applying forceps, ensure treatment plan is clear to all treating clinicians
	6	Thusu et al. 2012	National Patient Safety Database (NPSA) cross-sectional view of patient safety incidents (PSI), UK	Orthodontic PSIs account for 8.9% of all dental PSIs	1, 2	-
	7	Hebballi et al. 2015	Frequency and types of AEs associated with dental devices reported to the Food and Drug Administration and User Facility Device Experience (MAUDE) database, USA	Orthodontic appliances and accessories account for AEs in 1% of all dental devices AEs	1	-
	8	Hiivala et al. 2016	Incidence of dental patient safety incidents (PSIs) in hospital and private settings, Finland	Orthodontic PSIs account for 3.6% of all dental PSIs	1	-
	9	Bationo et al. 2016	In vitro investigation of monomer release from orthodontic adhesives using gas phase chromatography and mass spectrometry., Burkina Faso & France	Significant quantities of bisphenol A (BPA), triethylene glycol dimethacry-late (TEGDMA) and hydroxyethyl-methacrylate (HEMA) and other monomers were detected in orthodontic adhesives used in daily practice	1	-
Risk assessment of orthodontic procedures or materials	10	Abbott. 2000	Review of dental radiation, its effect on human tissues, risks and radiation protection, Australia	Recommendations to improve safety in dental radiography	2,3	Modern equipment in a well-designed surgery with sufficient wall thickness, good radiographic technique and compliance with the ALARA principle
	11	Chausu et al. 2000	Review of safe orthodontic bracket bonding in patients under general anaesthesia (GA), Israel	Recommendations on safe bracket bonding in patients under GA	2, 3	The use of rubber dam is highly recommended as it can facilitate safe and reliable bonding of brackets in disabled children under GA
	12	Samuels and Brezniak 2002	Orthodontic Facebows: safety issues and management, UK & Israel	Recommendations to improve Facebow safety	2, 3	A locking facebow with self-releasing head strap is indicated. Clear written instructions for proper use, removal and emergencies should be given to the patients
	13	Kravitz and Kusnoto 2007	Risks of orthodontic mini-implants, USA	Highlights clinical risks when using mini-implants while identifying solutions	2, 3	Suggestions to overcome risks during mini-implant insertion and orthodontic loading, soft tissue problems and complications during removal
	14	Kravitz and Kusnoto 2008	Risks of soft-tissue lasers in Orthodontics, USA	Clinical overview of the risks of soft-tissue lasers	2, 3	Clinicians need to obtain certification and train their stuff. Proper eye wear should be worn by the patient and the clinician/assistant, informed consent is needed and post-operative instructions should be given to the patient
	15	Johal et al. 2013	Prospective cohort study investigating effect of fixed appliance treatment on patient's diet, comparing with controls, UK	Fixed appliance treatment does not significantly affect energy or macro-nutrient intake, body mass index or body fat percentage	2	-
	16	Suzuki et al. 2013	Review of 186 mini-implants investigating the causes leading to failures, Japan	Mini-implants suggested minimum length is 5 mm for the maxilla and 6 mm for the mandible. Root proximity rather than bone density and insertion torque higher than 10Ncm are major factors in failures	2, 3	To avoid mini-implant failures, clinicians should use over 5-6 mm length screws, avoid root proximity and keep the insertion torque at fairly low levels
	17	McCusker et al. 2013	In vitro, eye safety of light curing lamps, UK	Low short term risks from plasma, halogen or LED curing lights, particularly if eye safety precautions are employed	2	-
	18	Kuroda and Tanaka 2014	Review of risks and complications of mini-implants, Japan	Factors leading to failures identified: age, smoking, oral hygiene, implant site, non-keratinized tissue, cortical bone thickness, bone density, root proximity, screw dimensions, insertion torque, loading and direction	2	-
	19	Görgülü et al. 2014	In Vitro, safety of metal fixed appliance during MRI, Turkey	Temperature changes considered to be in acceptable ranges. Brackets seem to be safe during an MRI, however it would be safe to remove the wires	2	-
	20	Mouhat 2017	In vitro, pulp chamber temperature with various types of LED curing units and different exposure times, distance and enamel thickness. Norway	Shorter distance, longer exposure times and thin enamel lead to higher pulp chamber temperatures	2	-
	21	Anwar and Waring 2017	Wrong tooth extraction in orthodontics: audit of extraction letters, UK	No occurrence of wrong site tooth extraction in this audit of 80 random extraction letters. Long term re-audits recommended to improve standard and prevent AEs	2	-
	22	Cullingham et al. 2017	Factors increasing risk of wrong tooth extraction and possible solutions to minimise the risk, UK	Highlights factors that increase the risk of wrong tooth extraction and identifies possible interventions to minimise the risk	2, 3	Treatment plan should be reasonable and clear from the referring clinician who also needs to communicate the requested tooth extraction with the use of two different charting methods. Increased awareness of the factors that predispose to wrong tooth extraction
	23	Jacob et al. 2021	Preventing wrong tooth extraction. Creation of a surgical checklist to reduce the risk, UK	Highlights the importance of checklists in preventing wrong site extraction by re-auditing clinical notes and checklist completion fortnightly for ten weeks	3	A computerised safety checklist for tooth extraction in primary care has the potential to improve patient safety by adopting measures to prevent wrong tooth extraction and standardising communication between clinicians
	24	Jerrold and Danoff-Rudick 2022	Study identifying never events in orthodontic care by the use of the Delphi protocol in form of questionnaires sent to orthodontists	Twelve never events identified with agreement > 95% amongst all orthodontists	2	
	25	Knoedler et al. 2023	Orthognathic surgery risk factors, USA & Germany	Identifies risk factors that can lead to pre- and postoperative complications	1, 2	-

The remaining sixteen studies reported risk assessments of orthodontic procedures or materials. These included safety assessment of dental radiography [23], bonding of brackets under general anaesthesia [24], orthodontic facebows [10], mini-implants [12, 25, 26], soft-tissue lasers in orthodontics [13], effect of orthodontic treatment on patients’ diet [14], eye safety of curing lights [27], safety of metal fixed appliance during magnetic resonance imaging (MRI) [28], pulp safety of various types of curing lights [29], wrong tooth extraction in orthodontics [30–32], orthodontic treatment by identifying orthodontic never events [33] and complications after orthognathic surgery [34]. These studies identified risks in orthodontic procedures or materials and proposed solutions to manage and minimise these risks.

### Study results

#### Measuring harm

Seven of the studies included provided information in the first category of the WHO’s research cycle on patient safety, which is “measuring harm” [4, 7–9, 19, 22, 34]. Sims et al. conducted a postal survey on eye protection in the UK and found that ocular injuries were reported in 37.7% of all respondents involving orthodontists, assistants and patients [19]. Peleg et al. conducted a root-cause analysis of wrong-tooth extraction in 54 insurance claims in Israel and reported that in two thirds of all claims an identification error was the cause of the incorrect tooth extraction [4]. Also, a cross-sectional study on PSIs in the UK found that orthodontic PSIs accounted for 8.9% of all reported dental PSIs in the country [7]. Hebballi et al. investigated the frequency and types of AEs associated with dental devices as reported to the Food and Drug Administration and User Facility Device Experience (MAUDE) in the US [9]. They reported that orthodontic appliances and accessories accounted for 1% of all AEs involving dental devices. In a similar investigation in hospital and private settings in Finland, Hiivala et al. reported that orthodontic PSIs accounted for 3.6% of all dental PSIs [8]. Finally, a multi-centre retrospective review of orthognathic surgeries assessing complications and risk factors studied a population of 674 patients [34]. They reported that adverse events were rare (4.3%) with superficial incisional infection being the most common. They also concluded that the setting, the type of surgery as well as the patients’ ethnicity were identified as risk factors for some types of complications.

#### Understanding causes of harm & identifying solutions

Twenty-one of the included studies identified the underlying causes of AEs that caused patient harm (WHO’s Category 2 “Understanding the causes”) [3, 4, 7, 10, 12–14, 19–21, 23–31, 33, 34]. In addition, twelve studies identified possible solutions that may be effective in reducing the occurrence of AEs (WHO’s Cycle Category 3 “Identifying solutions”) [4, 10, 12, 13, 19–21, 23–25, 31, 32]. These solutions included: health and safety instructions for eye-protection goggles to prevent ocular trauma [19], use of non-latex materials [21], clear instructions with a brief description of the tooth to be extracted addressed to the clinician using two different identification methods to prevent wrong-site extraction and use of a computerised checklist [4, 31, 32], use of facebows with a locking mechanism and self-releasing head strap to prevent injuries from headgear [10, 20], suggestions to improve safety in dental radiography [23], use of rubber dam during bonding of brackets under general anaesthesia [24], recommendations to overcome failures and risks during placement, loading and removal of mini-implants [12, 25] and, finally, instructions for safe use of soft-tissue lasers in orthodontics recommending that the clinician obtained appropriate training and certification, use of proper eye wear by all involved parties, obtaining informed consent and providing proper post-operative instructions [13].

None of the included studies provided information on how to evaluate the impact of such solutions or on how to translate evidence into safer care in terms of affordability and acceptability. Data synthesis and meta-analysis was not possible due to the heterogeneity of the different studies and the nature of the data.

## Discussion

### Patient safety incidents in orthodontics

To our knowledge, this is the first systematic investigation of patient safety in orthodontics. The lack of evidence in the field manifests in our results. Twenty-five studies were included in this review and these studies were only peripherally related to orthodontics while providing some information based on the WHO’s research cycle. This cycle describes a process to identify solutions for enhancing patient safety and reducing patient harm. It consists of five steps representing the natural process for patient-safety initiatives. It seems that dentistry in general and orthodontics in particular have yet to take even the initial steps of the cycle (steps 1 and 2), which are to measure the harm and understand the causes of harm [16]. This is evident from the results as the included studies were either reviews of risks associated with specific orthodontic procedures (like mini-implant insertion, soft-tissue laser, facebow use, etc.) or retrospective reviews of AEs peripherally related to orthodontics (incidence of ocular trauma, adverse reactions to materials, etc.).

The results of this review document that current evidence relating to orthodontics is scarce. Without a basic understanding of PSIs and harms we cannot begin to understand the causes and identify solutions that will subsequently translate into safer care for our patients [16]. A major limitation to this is a trend towards potential under-reporting of PSIs in our field. In fact, a review of the National Patient Safety Agency (NPSA) database in the UK revealed that orthodontics is among the lowest reporting specialties along with dental surgery and paediatric dentistry [35]. A contributing factor in this may be the lesser severity of some PSIs in orthodontics, which may be smaller injuries like soft-tissue laceration from loose wires [16]. One way to overcome the underreporting issues may be effective keeping of patient records and clinical notes, which may prove an essential tool in clinical audits and will also underpin the reporting of more AEs [36]. Also, the lack of standardisation in terminology and reporting process of AEs makes it challenging if not impossible to summarise and categorise all PSIs in orthodontics, let alone analyse the data in depth.

Additionally, we hypothesise that an underreporting bias may exist between dental specialities. Dental implants are more expensive and dentists and/or patients may therefore report them more often when asking for replacements [9]. This leads, e.g., to many more reported PSIs for implants than for burs. Finally, another contributing factor in the lack of evidence on patient safety is the overlap found in some areas within dentistry. This makes it more challenging to precisely measure AEs in only one field. A clear example of this is the AE of wrong-tooth extraction for orthodontic reasons, which may fall in both the orthodontic and surgical category.

### Standardisation and terminology

The lack of a standardised terminology and reporting of PSIs in orthodontics seems to hinder any effort to summarise and categorise PSIs, which could be a reasonable first research step to enhance our knowledge in this field. For future work in this field, we therefore suggest that PSIs related to orthodontics may be summarised into two main categories; local and systemic. Categorisation with subcategories and examples are shown in Table 2. Terminology according to the WHO is proposed in Table 3.

**Table 2:** Orthodontic patient safety incidents summarised as the two main categories of local and systemic, with examples. Local patient safety issues (PSIs) are related to harm in the dental (or surrounding) tissues, orofacial function and unwanted tooth movement. Systemic PSIs refer to pain and discomfort, emotional harm or other as part of an orthodontic treatment. Harms related to orthodontic care may fall into more than one of the categories

**A. LOCAL**	**Types of patient safety incidents (PSIs)**	**Examples**
	1. Dental tissues	Root resorption
		Pulp necrosis
		Caries
	2. Soft tissues	Gingival recessions
		Soft tissue laceration from loose wire
		Local allergic reaction/contact dermatitis
	3. Orofacial function	Development of lip catch
	4. Unwanted tooth movement	"Active" retainer with excessive incisor root torque movement out of the bone
**B. SYSTEMIC**	1. Pain and discomfort	Hypersensitivity due to excessive interproximal reduction
		Pain from a defective orthodontic appliance
	2. Emotional harm	Development of general discomfort/odontophobia associated with orthodontic treatment
		Development of mistrust towards clinician/health system in the context of orthodontic treatment
		Deterioration of one's own perception of oral health (oral health-related quality of life)
		Bullying as a result of delayed initiation of treatment due to delayed/inadequate diagnosis
	3. Other adverse effects	Inhalation of foreign object (orthodontic parts)
		Wrong-tooth extraction
		Cross-infection

**Table 3:** Terminology proposed in orthodontic patient safety future research inspired by the WHO’s Conceptual Framework for the International Classification for Patient Safety Final Technical Report

Term	Definition
Adverse event	An injury that was caused by medical management or complication instead of the underlying disease and that resulted in prolonged hospitalisation or disability at the time of discharge from medical care, or both. An event or omission arising during clinical care and causing physical or psychological injury to a patient
Cause	The act by which an effect is produced. An antecedent factor that contributes to an event, effect, result or outcome
Clinical audit	Organised review of clinical procedures/cycle of activities involving the measurement of care, comparison with a standard of some kind (whether process or outcome) and ideally interventions to improve quality where necessary
Clinical incident	Incidents in a health care setting caused by clinical procedures that resulted, or could have resulted, in unexpected harm to the patient
Complaint	An expression of dissatisfaction on the part of a patient that represents a particular perception of events. A complaint may or may not reveal that a mistake or an error has occurred
Critical incident	An incident resulting in serious harm to the patient when an evident need for immediate investigation and response exists
Harm	Temporary or permanent impairment of the physical, emotional or psychological function or structure of the body and/or pain resulting from the need for an intervention
Hazard	A situation or event that introduces or increases the probability of an adverse event. Potential source of harm
Iatrogenic	Injury or illness resulting from a diagnostic procedure, therapy, other element of healthcare or originating from or caused by a clinician including unintended or unnecessary harm
Medical error	An adverse event or near miss that is preventable with the current state of medical knowledge
Neglect	The absence of minimal services or resources to meet basic needs. Neglect may also include placing the individual in unsafe or unsupervised conditions
Patient safety	Freedom from accidental injury. The avoidance, prevention and amelioration of adverse outcomes or injuries stemming from the processes of healthcare. These events include “errors”, “deviations” and “accidents”. Safety emerges from the interaction of the components of the system; it does not reside in a person, device or department
Patient safety incident	An event or circumstance, which could have resulted, or did result in unnecessary harm to a patient
Prevention	Modification of the system to decrease the probability of the dreaded event arising and to return to an acceptable risk level; any measure aiming at reducing the frequency and the severity of the risk
Quality of care	Degree to which health services for individuals and populations increase the likelihood of desired health outcomes and are consistent with current professional knowledge
Risk	The likelihood, high or low, that somebody or something will be harmed by a hazard, multiplied by the severity of the potential harm
Safe care	Safe care involves making evidence-based clinical decisions to maximise the health outcomes of an individual and to minimise the potential for harm
Safety culture	An integrated pattern of individual and organisational behaviour, based upon shared beliefs and values, that continuously seeks to minimise patient harm that may result from the processes of care delivery
Underlying cause	The systems or process cause that allow for the proximate cause of an event to occur. Underlying causes may involve special-cause variation, common-cause variation, or both

Local PSIs refer to any harm on dental tissues (root resorption, white spot lesions, pulp necrosis, caries) and soft tissues. This may be damage to both periodontal and surrounding soft tissues that could have been avoided (gingival recessions, soft tissue lacerations, local allergic reaction/contact dermatitis). In addition, local PSIs include treatment injuries with a negative effect on orofacial function. This may be development of lip catch as a result of orthodontic treatment. Finally, any harm related to any unwanted tooth movement is also included in this category. This may be unwanted tooth movement due to an active retainer.

Systemic PSIs refer to harm at a systemic level. This may be excessive pain and discomfort as a result of the orthodontic treatment due to a defective appliance or even hypersensitivity due to excessive interproximal reduction. In addition, systemic PSIs include potential emotional damage to patients. This may be development of general discomfort/odontophobia/mistrust towards the clinician or the healthcare system or deterioration of the oral health-related quality of life (OHRQoL). Systemic PSIs may be a result of delayed treatment initiation due to delayed/inadequate diagnosis. Finally, harm caused by poor cross-infection control, inhalation of orthodontic parts and extraction of a wrong tooth are also considered systemic PSIs.

### Future research agenda

A proposal for a future research agenda in orthodontic patient safety is shown in Table 4. The agenda is intended as inspiration to promote future research and development in patient safety in orthodontics. It should not be considered absolute as topics other than those listed may be of interest for future patient safety initiatives. Two main categories of studies are presented in Table 4: Retrospective or prospective studies dealing with patient safety (26).

**Table 4:** Aspects and examples of future initiatives and research. Orthodontic patient safety research agenda should follow the WHO’s research cycle. The table does not represent all relevant aspects for future research. Rather, it highlights important aspects to elucidate among many other relevant aspects

The WHO's research cycle	Future research
1. Measuring harm	Retrospective reviews of all orthodontic PSIs from local or national agencies
	Prospective evaluation in selected clinics or with certain treatment modalities
2. Understanding causes	Root-cause analysis of PSI related to orthodontics using acknowledged tools
	Understanding patient harm from the patient's perspective
3. Identifying solutions	Local and general recommendations to prevent care
	Understanding the barriers that block harm prevention
	Learn and apply solutions from other part of the health care systems
4. Evaluating impact	National surveys
	Patient-centred studies
	Plan-Do-Act cycles
5. Translating evidence into safer care	Implement PS in the curriculum of dental schools/post-graduate orthodontic programs
	Apply evidence from other medical fields on how to promote safe patient care
	Encourage national and international orthodontic societies to play a vital role in the promotion of orthodontic care

Retrospective studies are reactive in nature and focus on the incidence, characteristics and severity of PSIs using an acknowledged methodology such as journal file audit and root cause analysis (RCA) (26,27). They investigate PSIs that have already occurred with the intention of generating knowledge to promote learning and guidance for future patient safety initiatives. RCA allows us to focus on individual PSIs and investigate, through a comprehensive analysis, all the contributing factors that lead to the occurrence of an AE.

Conversely, prospective studies assess potential risks associated with a treatment, appliance or material. The methodology in these studies is failure mode and effects analysis (FMEA) (27,28). This approach is the analysis of a method, treatment, material or procedure by first creating a risk map and then implementing measures to reduce the likelihood or impact of a PSI (27–30).

Both intrinsic and extrinsic motivation are key factors in the establishment of safer future orthodontic care. Intrinsic motivation is shaped by professional ethics, norms and patient-reported outcomes and expectations [1, 37, 38]. The articles included in our review, however, mainly focused on the extrinsic motivation, which refers to the environment, policies and strategies that we may develop with the ultimate goal of improving patient safety in orthodontics.

In orthodontic patient safety research, a need exists to increase our focus on this aspect and on clinical routines and administrative, organisational and legal contexts. One strategy that may help us move in this direction is to establish excellent records and clinical notes through periodical audits [30]. This will help clinicians and/or patients report more AEs in future. Honest exchange of such information between health professionals is a necessary first step and a founding rock for safer care and further research. To achieve this, it is important to establish a non-blame culture with psychological safety and a feeling of partnership, enthusiasm and commitment to improving patient safety in orthodontics [36].

Research on patient safety is more advanced in other parts of healthcare than orthodontics. Even other fields of dentistry have taken steps in this direction with the creation of checklists, i.e. in endodontics, orofacial function and oral surgery [39–43]. Checklists seem to have a positive effect on patient safety [44–46]. Most of the checklists are adaptations of the WHO’s surgical checklist that is now used in a wide range of surgical specialties in medicine [47]. Adjusting this to fit our orthodontic needs and implementing it in daily practice may be an important step towards improving safety in orthodontics [48]. In the past decade, the WHO has published several guidelines and educational curricula to enhance the level of patient safety in healthcare in general [49, 50]. These publications may provide a starting point for the spreading of local patient safety initiatives and the introduction of educational and organisational measures to further patient safety.

Some orthodontic societies seem to have taken steps towards patient safety, however all societies in different countries need to follow and implement policies for safer care. In its core patient safety is the purpose of audit and clinical governance. Amongst other, research is a vital element in this process. Nevertheless, a limitation in this could be that clinical governance might differ from one country to another.

Traditionally, patient safety was focused on rare types of incidents with a significant degree of harm referred to as “never events” in the literature [51]. However, in recent years, more efforts have been devoted to understanding the frequency and causes of PSIs that we assume occur more frequently than is reported today [51]. The perceived threshold determining what is considered a PSI may often be vague; and the border is not absolute, particularly as we come to understand patient safety better. It is important to emphasize that common side effects (e.g., root resorption) are not considered PSIs as these side effects may also occur when a patient has undergone an optimally performed course of treatment, unless, of course, these side effects were avoidable and appropriate measures had been adopted [52]. The extent of such side effects, however, can vary and probably depends on a wide range of factors (force magnitude, treatment duration) [53]. Excessive root resorption, however, may be considered a PSI if the risk factors were not assessed before initiating treatment and if precautionary measures were not taken in advance. A step towards safer orthodontics may be to incorporate such “risk maps” routinely in systematic reviews. For example, when a systematic review compares A to B, reporting just which of the two is more efficient or faster may be insufficient. The burden and the risk of harm to the patient should also be reported. This reporting may include anything that may be considered a PSI, from excessive root resorption to increased exposure to radiation, cytotoxicity, effect on patients’ OHRQoL, late diagnosis, overtreatment, gingival recessions or bone dehiscence, etc. A cultural change in the way we approach these “side effects” and further patient-centred research will improve patient safety in our field. In addition, in today's rapidly evolving technological landscape, where new advancements outpace research capabilities, emphasizing the safety of orthodontic materials is crucial while treatment decisions need to be patient-centred, based on their perspective [54].

### Strengths and limitations

The strengths of this systematic review include an extensive literature search, a predefined protocol, a priori registration with PROSPERO and the adoption of a strict methodology at all study stages [55]. Also, the fact that there was no date or language limitation in the search, provided us with data that likely reflect the current understanding and knowledge about PSI in orthodontics. In addition, the proposed categorisation of PSIs in orthodontics and the future-agenda proposals may spark interest and lead to further research in the field of orthodontic patient safety.

Certain limitations need further consideration: mainly the inability to assess precise prevalence of orthodontic PSIs and categorise them accordingly. This inability is due to the poor current evidence and lack of standardisation and terminology and the fact that many PSIs are probably underreported. It can also be due to the fact that patient safety is a topic of increasing complexity, especially with the new risks arising directly from the use of new technologies [51]. Also, there is inherent risk of bias due to the nature of the studies included which were mostly retrospective [56]. Furthermore, in this study, the final selection of the included studies was consensus-based instead of individually assessing the suitability of the articles during the review process. Finally, despite thorough searching, there could be studies overlooked during the process, possibly originating from databases not encompassed in the search.

## Conclusion

Current evidence on patient safety in orthodontics is scarce due to a lack of standardisation and potential under-reporting of PSIs. The current literature on orthodontic patient safety deals mostly with “measuring harms” and “understanding causes of patient safety”, whereas less attention has been devoted to initiatives “identifying solutions”, “evaluating impact” and “translating evidence into safer care”. The present project presents proposals for a new categorisation, terminology and a future research agenda that may serve as a framework to support future research and clinical initiatives to improve patient safety in orthodontic care.

### Supplementary Information

Supplementary Material 1.

Supplementary Material 2.

## Data Availability

All data generated or analysed during this study are included in this published article and its supplementary information files.
