# Supplementary material for: Patient safety in orthodontic care: a scoping literature review with proposal for terminology and future research agenda
Source: BMC Oral Health. 2024 Jun 18;24:702. doi: 10.1186/s12903-024-04375-7 (PMC11184803; doi:10.1186/s12903-024-04375-7)
Supplement: Supplementary file 1 — Supplementary Material 1. [file 12903_2024_4375_MOESM1_ESM.docx]

Supplementary Table 1. Studies excluded by abstract (n = 155)

|  | Title | Authors | Journal/Book | Status |
| --- | --- | --- | --- | --- |
| 1 | Unintended consequences of patient online access to health records: a qualitative study in UK primary care | Turner A, Morris R, McDonagh L, Hamilton F, Blake S, Farr M, Stevenson F, Banks J, Atherton H, Rakhra D, Lasseter G, Feder G, Ziebland S, Hyde E, Powell J, Horwood J. | Br J Gen Pract | Excluded by abstract |
| 2 | Perceptions of Patient Safety Competence Using the Modified Version of the Health Professional Education in Patient Safety Survey (H-PEPSS) Instrument Among Dental Students in Riyadh, Saudi Arabia | Halawany HS, Abraham NB, Al-Badr AH, Al-Khalifa KS. | Adv Med Educ Pract | Excluded by abstract |
| 3 | An unusual foreign body in the oral cavity: a case report from a patient safety point of view and literature review | Ito M, Watanabe N, Sawado Y, Ishida K, Yoshiyama Y, Ishida T, Fuseya S, Tanaka S, Kawamata M. | J Anesth | Excluded by abstract |
| 4 | Development of an Inventory of Dental Harms: Methods and Rationale | Kalenderian E, Lee JH, Obadan-Udoh EM, Yansane A, White JM, Walji MF. | J Patient Saf | Excluded by abstract |
| 5 | Foreign body aspiration and ingestion in dental clinic: a seven-year retrospective study | Huh J, Lee N, Kim KY, Jung S, Cha J, Kim KD, Park W. | J Dent Anesth Pain Med | Excluded by abstract |
| 6 | Early identification of malignancy in trismus: ten-year evolution of a trismus checklist to improve patient safety | Crawford CE, Srinivas A, Momin P, Watts J, Davies SJ, Pemberton MN. | Br Dent J | Excluded by abstract |
| 7 | Managing dental unit waterlines: a quality improvement programme | Umer F, Khan M, Khan FR, Tejani K. | BMJ Open Qual | Excluded by abstract |
| 8 | Effects of a Radiography Checklist on Reducing Retake Exposures | Nenad MW. | J Dent Hyg | Excluded by abstract |
| 9 | Patient safety in dentistry - the bigger picture | Chohan P, Renton T, Wong J, Bailey E. | Br Dent J | Excluded by abstract |
| 10 | Infection Prevention and Control in Dental Practice: Revised version adopted by the General Assembly 27-29 September 2021, Sydney, Australia. Revised version adopted by the General Assembly: September 2019, San Francisco, United States of America. Original version adopted by the General Assembly: September 2009, Singapore, Singapore | FDI | Int Dent J | Excluded by abstract |
| 11 | Adverse events during sedation for oro-dental trauma in an Israeli paediatric emergency department | Bilder L, Librov S, Gutmacher Z, Pasternak I, Shavit I. | Dent Traumatol | Excluded by abstract |
| 12 | Is it safe to place implants in patients at risk of MRONJ? | Owen B, Bradley H. | Evid Based Dent | Excluded by abstract |
| 13 | Implementation of teledentistry for orthodontic practices | Park JH, Kim JH, Rogowski L, Al Shami S, Howell SEI. | J World Fed Orthod | Excluded by abstract |
| 14 | Patient Safety in Dental Practice: Lessons to Learn About the Risks and Limits of Professional Liability | Corte-Real A, Caetano C, Alves S, Pereira AD, Rocha S, Nuno Vieira D. | Int Dent J | Excluded by abstract |
| 15 | Orthognathic surgery in COVID-19 times, is it safe? | Glen P, Aurora F, Thomas S, Kissun D. | Br J Oral Maxillofac Surg | Excluded by abstract |
| 16 | The application of a new clear removable appliance with an occlusal splint in early anterior crossbite | Zhang J, Yang Y, Han X, Lan T, Bi F, Qiao X, Guo W. | BMC Oral Health | Excluded by abstract |
| 17 | Quantitative measurements of aerosols from air-polishing and ultrasonic devices: (How) can we protect ourselves? | Kaufmann M, Solderer A, Gubler A, Wegehaupt FJ, Attin T, Schmidlin PR. | PLoS One | Excluded by abstract |
| 18 | Metal hypersensitivity screening among frontline healthcare workers-A descriptive study | Dordunoo D, Hass M, Smith C, Aviles-Granados ML, Weinzierl M, Anaman-Torgbor JA, Shaik A, Mallidou A, Adib F. | J Clin Nurs | Excluded by abstract |
| 19 | Incidence, nature and causes of avoidable significant harm in primary care in England: retrospective case note review | Avery AJ, Sheehan C, Bell B, Armstrong S, Ashcroft DM, Boyd MJ, Chuter A, Cooper A, Donnelly A, Edwards A, Evans HP, Hellard S, Lymn J, Mehta R, Rodgers S, Sheikh A, Smith P, Williams H, Campbell SM, Carson-Stevens A. | BMJ Qual Saf | Excluded by abstract |
| 20 | Overtreatment as an ethical dilemma in Australian private dentistry: A qualitative exploration | Holden ACL, Adam L, Thomson WM. | Community Dent Oral Epidemiol | Excluded by abstract |
| 21 | Patient safety culture amongst dental students and interns in Dammam, Saudi Arabia | AlOlayan R, Alahmad A, Buali D, Alonaizan F, Alhareky M, Alhumaid J, Nazir MA. | Eur J Dent Educ | Excluded by abstract |
| 22 | Use of Laser Systems in Orthodontics | Demirsoy KK, Kurt G. | Turk J Orthod | Excluded by abstract |
| 23 | Perspectives of tele-orthodontics in the COVID-19 emergency and as a future tool in daily practice | Saccomanno S, Quinzi V, Sarhan S, Laganà D, Marzo G. | Eur J Paediatr Dent | Excluded by abstract |
| 24 | Patient safety in primary and outpatient health care | Kuriakose R, Aggarwal A, Sohi RK, Goel R, Rashmi NC, Gambhir RS. | J Family Med Prim Care | Excluded by abstract |
| 25 | Beyond 'find and fix': improving quality and safety through resilient healthcare systems | Anderson JE, Ross AJ, Back J, Duncan M, Snell P, Hopper A, Jaye P. | Int J Qual Health Care | Excluded by abstract |
| 26 | Dentists' practices and patient safety: A cross-sectional study | Al-Mahalawy H, El-Mahallawy Y, El Tantawi M. | Eur J Dent Educ | Excluded by abstract |
| 27 | Patients' and healthcare workers' recommendations for a surgical patient safety checklist - a qualitative study | Harris K, Søfteland E, Moi AL, Harthug S, Storesund A, Jesuthasan S, Sevdalis N, Haugen AS. | BMC Health Serv Res | Excluded by abstract |
| 28 | Assessing the Patient Safety Culture in Dentistry | Yansane A, Lee JH, Hebballi N, Obadan-Udoh E, White J, Walji M, Easterday C, Rindal B, Worley D, Kalenderian E. | JDR Clin Trans Res | Excluded by abstract |
| 29 | Comparison of Orthognathic Surgery Outcomes Between Patients With and Without Underlying High-Risk Conditions: A Multidisciplinary Team-Based Approach and Practical Guidelines | Chou PY, Denadai R, Chen C, Pai BC, Hsu KH, Chang CT, Pascasio D, Lin JA, Chen YR, Lo LJ. | J Clin Med | Excluded by abstract |
| 30 | Lingual frenuloplasty with myofunctional therapy: Exploring safety and efficacy in 348 cases | Zaghi S, Valcu-Pinkerton S, Jabara M, Norouz-Knutsen L, Govardhan C, Moeller J, Sinkus V, Thorsen RS, Downing V, Camacho M, Yoon A, Hang WM, Hockel B, Guilleminault C, Liu SY. | Laryngoscope Investig Otolaryngol | Excluded by abstract |
| 31 | Medication Safety: Reducing Anesthesia Medication Errors and Adverse Drug Events in Dentistry Part 1 | Sarasin DS, Brady JW, Stevens RL. | Anesth Prog | Excluded by abstract |
| 32 | Factors affecting patient safety culture among dental healthcare workers: A nationwide cross-sectional survey | Cheng HC, Yen AM, Lee YH. | J Dent Sci | Excluded by abstract |
| 33 | Effectiveness of a clinical guideline to improve dental health among orthodontically treated patients: study protocol for a cluster randomized controlled trial | Oosterkamp BC, Wafae A, Schols JG, van der Sanden WJ, Wensing M. | Trials | Excluded by abstract |
| 34 | Safety in office-based anesthesia: an updated review of the literature from 2016 to 2019 | de Lima A, Osman BM, Shapiro FE. | Curr Opin Anaesthesiol | Excluded by abstract |
| 35 | Preventable patient harm is expensive | Hellyer P. | Br Dent J | Excluded by abstract |
| 36 | Dental Patient Safety in the Military Health System: Joining Medicine in the Journey to High Reliability | Stahl JM, Mack K, Cebula S, Gillingham BL. | Mil Med | Excluded by abstract |
| 37 | Minimum size and positioning of imaging field for CBCT scans of impacted maxillary canines | Pakbaznejad Esmaeili E, Ilo AM, Waltimo-Sirén J, Ekholm M. | Clin Oral Investig | Excluded by abstract |
| 38 | Relationships between dental hygienists' work environment and patient safety culture | Choi EM, Mun SJ, Chung WG, Noh HJ. | BMC Health Serv Res | Excluded by abstract |
| 39 | Twenty four years of oral and maxillofacial surgery malpractice claims in Spain: patient safety lessons to learn | Bordonaba-Leiva S, Gómez-Durán EL, Balibrea JM, Benet-Travé J, Martin-Fumadó C, Bescos Atin C, Mareque-Bueno J, Arimany-Manso J. | Oral Maxillofac Surg | Excluded by abstract |
| 40 | A comparison of patient safety culture at two campuses of Riyadh based dental College | Ahsan SH, Abdul Wahid AQ, Alali S, ALlqadhi A, Alnakly Z, Al-Turki M, Hassan MA, Almarshd A. | J Pak Med Assoc | Excluded by abstract |
| 41 | Effect of loaded orthodontic miniscrew implant on compressive stresses in adjacent periodontal ligament | Albogha MH, Takahashi I. | Angle Orthod | Excluded by abstract |
| 42 | Polymer-based dental filling materials placed during pregnancy and risk to the foetus | Berge TLL, Lygre GB, Lie SA, Björkman L. | BMC Oral Health | Excluded by abstract |
| 43 | Beyond 'health and safety' - the challenges facing students asked to work outside of their comfort, qualification level or expertise on medical elective placement | Wiskin C, Dowell J, Hale C. | BMC Med Ethics | Excluded by abstract |
| 44 | A study on the efficacy and safety of combining dental surgery with tonsillectomy in pediatrics | Syed F, Uffman JC, Tumin D, Flaitz CM, Tobias JD, Raman VT. | Clin Cosmet Investig Dent | Excluded by abstract |
| 45 | Patient safety culture perceptions in the college of dentistry | Al Sweleh FS, Al Saedan AM, Al Dayel OA. | Medicine (Baltimore) | Excluded by abstract |
| 46 | A Patient Safety Toolkit for Family Practices | Campbell SM, Bell BG, Marsden K, Spencer R, Kadam U, Perryman K, Rodgers S, Litchfield I, Reeves D, Chuter A, Doos L, Ricci-Cabello I, Gill P, Esmail A, Greenfield S, Slight S, Middleton K, Barnett J, Moore M, Valderas JM, Sheikh A, Avery AJ. | J Patient Saf | Excluded by abstract |
| 47 | Incident reporting in dentistry: Clinical supervisor's awareness, practice and perceived barriers | AlBlaihed RM, AlSaeed MI, Abuabat AA, Ahsan SH. | Eur J Dent Educ | Excluded by abstract |
| 48 | Patient safety: Swallowed objects | Argent VA. | Br Dent J | Excluded by abstract |
| 49 | Dentists Survey on Adverse Events During Their Clinical Training | Osegueda-Espinosa AA, Sánchez-Pérez L, Perea-Pérez B, Labajo-González E, Acosta-Gio AE. | J Patient Saf | Excluded by abstract |
| 50 | Office-Based Anesthesia: Safety and Outcomes in Pediatric Dental Patients | Spera AL, Saxen MA, Yepes JF, Jones JE, Sanders BJ. | Anesth Prog | Excluded by abstract |
| 51 | Patient Assessment | Miloro M, Basi D, Halpern L, Kang D. | J Oral Maxillofac Surg | Excluded by abstract |
| 52 | Patients' evaluations of patient safety in English general practices: a cross-sectional study | Ricci-Cabello I, Marsden KS, Avery AJ, Bell BG, Kadam UT, Reeves D, Slight SP, Perryman K, Barnett J, Litchfield I, Thomas S, Campbell SM, Doos L, Esmail A, Valderas JM. | Br J Gen Pract | Excluded by abstract |
| 53 | Patient safety in dentistry: development of a candidate 'never event' list for primary care | Black I, Bowie P. | Br Dent J | Excluded by abstract |
| 54 | Patient safety: Needle breakage | Makwana M, Walsh S. | Br Dent J | Excluded by abstract |
| 55 | Review of never and serious events related to dentistry 2005-2014 | Renton T, Sabbah W. | Br Dent J | Excluded by abstract |
| 56 | In vitro and in vivo evidence of the cytotoxic and genotoxic effects of metal ions released by orthodontic appliances: A review | Martín-Cameán A, Jos Á, Mellado-García P, Iglesias-Linares A, Solano E, Cameán AM. | Environ Toxicol Pharmacol | Excluded by abstract |
| 57 | Surgical safety checklists and understanding of Never Events, in UK and Irish dental hospitals | Pemberton MN. | Br Dent J | Excluded by abstract |
| 58 | Patient Safety Challenges in General Dentistry | Hester DO. | Todays FDA | Excluded by abstract |
| 59 | Postoperative Adverse Events Inconsistently Improved by the World Health Organization Surgical Safety Checklist: A Systematic Literature Review of 25 Studies | de Jager E, McKenna C, Bartlett L, Gunnarsson R, Ho YH. | World J Surg | Excluded by abstract |
| 60 | Experimental investigation of the fracture torque of orthodontic anchorage screws | Reimann S, Ayubi M, McDonald F, Bourauel C. | J Orofac Orthop | Excluded by abstract |
| 61 | Contemporary views of dental practitioners' on patient safety | Bailey E. | Br Dent J | Excluded by abstract |
| 62 | Systematic review of patient safety interventions in dentistry | Bailey E, Tickle M, Campbell S, O'Malley L. | BMC Oral Health | Excluded by abstract |
| 63 | Reducing Harm in Healthcare Systems | O'Brien T. | Prim Dent J | Excluded by abstract |
| 64 | Litigation and Legislation. Risk management strategies in orthodontics. Part 2: Administrative considerations | Abdelkarim A, Jerrold L. | Am J Orthod Dentofacial Orthop | Excluded by abstract |
| 65 | Developing and Implementing a Culture of Safety in the Dentoalveolar Surgical Practice | Hupp JR. | Oral Maxillofac Surg Clin North Am | Excluded by abstract |
| 66 | Can patients detect hazardous dental practice? A patient complaint study | Hiivala N, Mussalo-Rauhamaa H, Murtomaa H. | Int J Health Care Qual Assur | Excluded by abstract |
| 67 | Radiological protection in computed tomography and cone beam computed tomography | Rehani MM. | Ann ICRP | Excluded by abstract |
| 68 | Adverse events in Public Dental Service in a Swedish county--a survey of reported cases over two years | Jonsson L, Gabre P. | Swed Dent J | Excluded by abstract |
| 69 | Preventing wrong tooth extraction: experience in development and implementation of an outpatient safety checklist | Saksena A, Pemberton MN, Shaw A, Dickson S, Ashley MP. | Br Dent J | Excluded by abstract |
| 70 | Exploring patient safety culture in primary care | Verbakel NJ, Van Melle M, Langelaan M, Verheij TJ, Wagner C, Zwart DL. | Int J Qual Health Care | Excluded by abstract |
| 71 | Over-the-counter (OTC) bruxism splints available on the Internet | Wassell RW, Verhees L, Lawrence K, Davies S, Lobbezoo F. | Br Dent J | Excluded by abstract |
| 72 | TADs: an evolutionary road to success | Pace A, Sandler J. | Dent Update | Excluded by abstract |
| 73 | Analysis of the attitudes and needs/demands of dental practitioners in the field of patient safety and risk management | Yamalik N, Van Dijk W. | Int Dent J | Excluded by abstract |
| 74 | [The application of air abrasion in dentistry] | Mandinić Z, Vulićević ZR, Beloica M, Radović I, Mandić J, Carević M, Tekić J. | Srp Arh Celok Lek | Excluded by abstract |
| 75 | Integrating Patient Safety in the OMFS Curriculum: Survey of 4-Year Residency Programs | Buhrow SM, Buhrow JA. | J Patient Saf | Excluded by abstract |
| 76 | Reducing oral and maxillofacial surgery resident risk exposure: lessons from graduate medical education reform | Buhrow SM, Buhrow JA. | J Dent Educ | Excluded by abstract |
| 77 | Patient safety incident prevention and management among Finnish dentists | Hiivala N, Mussalo-Rauhamaa H, Murtomaa H. | Acta Odontol Scand | Excluded by abstract |
| 78 | Safety issues of tooth whitening using peroxide-based materials | Li Y, Greenwall L. | Br Dent J | Excluded by abstract |
| 79 | An adverse event trigger tool in dentistry: a new methodology for measuring harm in the dental office | Kalenderian E, Walji MF, Tavares A, Ramoni RB. | J Am Dent Assoc | Excluded by abstract |
| 80 | First, do no harm | Cuny EJ; Organization for Safety, Asepsis and Prevention. | J Am Dent Assoc | Excluded by abstract |
| 81 | Patient safety incidents reported by Finnish dentists; results from an internet-based survey | Hiivala N, Mussalo-Rauhamaa H, Murtomaa H. | Acta Odontol Scand | Excluded by abstract |
| 82 | Patient safety and dentistry: what do we need to know? Fundamentals of patient safety, the safety culture and implementation of patient safety measures in dental practice | Yamalik N, Perea Pérez B. | Int Dent J | Excluded by abstract |
| 83 | Are health professionals' perceptions of patient safety related to figures on safety incidents? | Martijn L, Harmsen M, Gaal S, Mettes D, van Dulmen S, Wensing M. | J Eval Clin Pract | Excluded by abstract |
| 84 | A five-year assessment of clinical incidents requiring transfer in a dental hospital day surgery unit | Verco S, Bajurnow A, Grubor D, Chandu A. | Aust Dent J | Excluded by abstract |
| 85 | [Patient safety in general dental practice 2009] | Mettes TG, Bruers JM, van der Sanden WJ, van Eeten Kruiskamp L, van der Horst RH, Harmsen M, Wensing M. | Ned Tijdschr Tandheelkd | Excluded by abstract |
| 86 | Patient safety in dentistry: dental care risk management plan | Perea-Pérez B, Santiago-Sáez A, García-Marín F, Labajo-González E, Villa-Vigil A. | Med Oral Patol Oral Cir Bucal | Excluded by abstract |
| 87 | Patient safety in Dutch primary care: a study protocol | Harmsen M, Gaal S, van Dulmen S, de Feijter E, Giesen P, Jacobs A, Martijn L, Mettes T, Verstappen W, Nijhuis-van der Sanden R, Wensing M. | Implement Sci | Excluded by abstract |
| 88 | Guided tissue regeneration and orthodontics. A review of the literature | Reichert C, Deschner J, Kasaj A, Jäger A. | J Orofac Orthop | Excluded by abstract |
| 89 | Dental patient safety | Sjelin R. | Dent Assist | Excluded by abstract |
| 90 | Improving patient safety | Touati B. | Pract Proced Aesthet Dent | Excluded by abstract |
| 91 | [Bioethics and biosafety: the use of biomaterials in dental practice] | Bugarin Júnior JG, Garrafa V. | Rev Saude Publica | Excluded by abstract |
| 92 | Processing of dental instruments--are you following the correct procedures to ensure that your instruments are safe for patient treatment?--part II | Lassiter TE, Conte M. | J N J Dent Assoc | Excluded by abstract |
| 93 | Antimicrobial mouthrinse as part of a comprehensive oral care regimen. Safety and compliance factors | Silverman S Jr, Wilder R. | J Am Dent Assoc | Excluded by abstract |
| 94 | Wrong-side/wrong-site, wrong-procedure, and wrong-patient adverse events: Are they preventable? | Seiden SC, Barach P. | Arch Surg | Excluded by abstract |
| 95 | The safety and efficacy of treatment with air abrasion technology | Wright GZ, Hatibovic-Kofman S, Millenaar DW, Braverman I. | Int J Paediatr Dent | Excluded by abstract |
| 96 | Caries diagnosis and risk assessment. A review of preventive strategies and management | (no authors listed) | J Am Dent Assoc | Excluded by abstract |
| 97 | An update on dental radiology: quality and safety | Bridgman JB, Campbell DJ. | N Z Dent J | Excluded by abstract |
| 98 | Aspiration of foreign materials in children while under general anesthesia for dental extractions | Davis J, Anaes FC, Alton H, Butler J. | Anesth Pain Control Dent | Excluded by abstract |
| 99 | Radiation safety for children | White SC. | Int Dent J | Excluded by abstract |
| 100 | Patient safety during dental X-ray procedures | Gibbs SJ. | J Tenn Dent Assoc | Excluded by abstract |
| 101 | Adverse events in orthodontic practice: a review of the literature | Vaquero-Nino, P.; Perea-Perez, B.; Labajo-Gonzalez, E.; Santiago-Saez, A. | DENTAL CADMOS | Excluded by abstract |
| 102 | Dental injuries in younger emergency department patients | James, Vigil; Vandersluis, Yona R.; Zhang, Evangeline W. J.; Scolnik, Dennis | CANADIAN JOURNAL OF EMERGENCY MEDICINE | Excluded by abstract |
| 103 | A randomized trial on the effects of root resorption after orthodontic treatment using pulsating force | Wang, Jue; Lamani, Ejvis; Christou, Terpsithea; Li, Peng; Kau, Chung How | BMC ORAL HEALTH | Excluded by abstract |
| 104 | Effectiveness of a clinical guideline to improve dental health among orthodontically treated patients: study protocol for a cluster randomized controlled trial | Oosterkamp, Barbara C. M.; Wafae, Afzal; Schols, Jan G. J. H.; van der Sanden, Wil J. M.; Wensing, Michel | TRIALS | Excluded by abstract |
| 105 | The need for better studies to assess the safety of providing dental care for medically complex patients | Glick, Michael | JOURNAL OF THE AMERICAN DENTAL ASSOCIATION | Excluded by abstract |
| 106 | Lasers in dentistry | WIGDOR, HA; WALSH, JT; FEATHERSTONE, JDB; VISURI, SR; FRIED, D; WALDVOGEL, JL | LASERS IN SURGERY AND MEDICINE | Excluded by abstract |
| 107 | The Application of Air Abrasion in Dentistry | Mandinic, Zoran; Vulicevic, Zoran R.; Beloica, Milos; Radovic, Ivana; Mandic, Jelena; Carevic, Momir; Tekic, Jasmina | SRPSKI ARHIV ZA CELOKUPNO LEKARSTVO | Excluded by abstract |
| 108 | Bimaxillary Orthognathic Surgery Is Associated With an Increased Risk of Early Complications | Kantar, Rami S.; Cammarata, Michael J.; Rifkin, William J.; Alfonso, Allyson R.; DeMitchell-Rodriguez, Evellyn M.; Noel, Daphney Y.; Greenfield, Jason A.; Levy-Lambert, Dina; Rodriguez, Eduardo D. | JOURNAL OF CRANIOFACIAL SURGERY | Excluded by abstract |
| 109 | Lasers in dentistry: medico-legal problems and implications | Rini, Maria Sofia; Bucci, Marco Brady; Marini, Maurizio; Bucci, Dario Brady; D'Urso, Diego | DENTAL CADMOS | Excluded by abstract |
| 110 | The value of checklists | Tokede O, Ramoni R, Kalenderian E. | J Am Dent Assoc | Excluded by abstract |
| 111 | Lasers in Dentistry | MIDDA, M; RENTONHARPER, P | BRITISH DENTAL JOURNAL | Excluded by abstract |
| 112 | Efficacy and safety of piezocision in accelerating maxillary anterior teeth en-masse retraction: study protocol for a randomized controlled trial | Xu, Yichen; Yu, Liming; Tong, Xianqin; Wang, Yuhui; Li, Yuanyuan; Pan, Jie; Yang, Yanjing; Liu, Yuehua | TRIALS | Excluded by abstract |
| 113 | Safety and Effects of the Rapid Maxillary Expander on Temporomandibular Joint in Subjects Affected by Juvenile Idiopathic Arthritis: A Retrospective Study | Abate, Andrea; Cavagnetto, Davide; Rusconi, Francesca Maria Emilia; Cressoni, Paolo; Esposito, Luca | CHILDREN-BASEL | Excluded by abstract |
| 114 | Effect of vibration on orthodontic tooth movement in a double blind prospective randomized controlled trial | Mayama, Atsushi; Seiryu, Masahiro; Takano-Yamamoto, Teruko | SCIENTIFIC REPORTS | Excluded by abstract |
| 115 | Efficacy and safety of different interventions to accelerate maxillary canine retraction following premolar extraction: A systematic review and network meta-analysis | MacDonald, Laura; Zanjir, Maryam; Lighvan, Nima Laghapour; da Costa, Bruno R.; Suri, Sunjay; Azarpazhooh, Amir | ORTHODONTICS & CRANIOFACIAL RESEARCH | Excluded by abstract |
| 116 | Efficacy and safety of diode laser during circumvestibular incision for Le Fort I osteotomy in orthognathic surgery: a triple-blind randomized clinical trial | Jaeger, Filipe; de Oliveira Chiavaioli, Gustavo Marques; de Toledo, Guilherme Lacerda; Freire-Maia, Belini; Figueiredo Amaral, Marcio Bruno; Nogueira Guimaraes de Abreu, Mauro Henrique; Almeida de Arruda, Jose Alcides; Mesquita, Ricardo Alves | LASERS IN MEDICAL SCIENCE | Excluded by abstract |
| 117 | Management and prevention of gingival recession | Merijohn, George K. | PERIODONTOLOGY 2000 | Excluded by abstract |
| 118 | Computerized navigation surgery for the safe placement of palatal implants | Wexler, Alon; Tzadok, Sasi; Casap, Nardy | AMERICAN JOURNAL OF ORTHODONTICS AND DENTOFACIAL ORTHOPEDICS | Excluded by abstract |
| 119 | Miniscrew-assisted nonsurgical palatal expansion before orthognathic surgery for a patient with severe mandibular prognathism | Lee, Kee-Joon; Park, Young-Chel; Park, Joo-Young; Hwang, Woo-Sang | AMERICAN JOURNAL OF ORTHODONTICS AND DENTOFACIAL ORTHOPEDICS | Excluded by abstract |
| 120 | Combined Primary Cleft Lip and Palate Repair: Is It Safe? | Kantar, Rami S.; Rifkin, William J.; Cammarata, Michael J.; Maliha, Samantha G.; Diaz-Siso, J. Rodrigo; Farber, Scott J.; Flores, Roberto L. | JOURNAL OF CRANIOFACIAL SURGERY | Excluded by abstract |
| 121 | Tomographic mapping of buccal shelf area for optimum placement of bone screws: A three-dimensional cone-beam computed tomography evaluation | Kolge, Neeraj Eknath; Patni, Vivek J.; Potnis, Sheetal S. | APOS TRENDS IN ORTHODONTICS | Excluded by abstract |
| 122 | Is It Safe to Re-Harvest the Anterior Iliac Crest to Manage Le Fort I Interpositional Defects in Young Adults With a Repaired Cleft? | Posnick, Jeffrey C.; Gray, James A. | JOURNAL OF ORAL AND MAXILLOFACIAL SURGERY | Excluded by abstract |
| 123 | The efficacy of combination analgesic therapy in relieving dental pain | Mehlisch DR. | J Am Dent Assoc | Excluded by abstract |
| 124 | Extraction or preservation of deciduous molars in early mixed dentition as an interceptive treatment in agenesis of mandibular premolars in normal occlusion: A systematic review | Naoumova, Julia; Arcidiacono, Stefano; Hansen, Ken; Liljegren, Ann; Sjoegren, Petteri | JOURNAL OF THE WORLD FEDERATION OF ORTHODONTISTS | Excluded by abstract |
| 125 | Food allergens in oral care products | Coimbra, L; Costa, IM; Evangelista, JG; Figueiredo, A | SCIENTIFIC REPORTS | Excluded by abstract |
| 126 | Optimal sites for orthodontic anchor screw placement using panoramic images: risk of maxillary sinus perforation and contact with adjacent tooth roots during screw placement | Miyazawa, Ken; Shibata, Momoko; Tabuchi, Masako; Kawaguchi, Misuzu; Shimura, Noriko; Goto, Shigemi | PROGRESS IN ORTHODONTICS | Excluded by abstract |
| 127 | Patient safety with orthognathic surgery in an outpatient setting | Pekkari C, Weiner CK, Marcusson A, Davidson T, Naimi-Akbar A, Lund B. | Int J Oral Maxillofac Surg. 2023 Jul;52(7):806-812. doi: 10.1016/j.ijom.2022.12.001. Epub 2022 Dec 15. | Excluded by abstract |
| 128 | Diagnostic errors in Dentistry, opinions of egyptian dental teaching staff, a cross-sectional study | El-Wakeel N, Ezzeldin N. | BMC Oral Health. 2022 Dec 20;22(1):621. doi: 10.1186/s12903-022-02565-9. | Excluded by abstract |
| 129 | Allergic reactions to local anesthetic mepivacaine in dental procedures: a case report | Nam Y, Min S, Park W, Kim KD. | J Dent Anesth Pain Med. 2023 Jun;23(3):173-177. doi: 10.17245/jdapm.2023.23.3.173. Epub 2023 May 26. | Excluded by abstract |
| 130 | Droplet size distribution, atomization mechanism and dynamics of dental aerosols | Kayahan, E; Wu, M; Van Gerven, T; Braeken, L; Stijven, L; Politis, C; Leblebici, ME | JOURNAL OF AEROSOL SCIENCE | Excluded by abstract |
| 131 | Surgical safety checklists for dental implant surgeries-a scoping review | Kupka, JR; Sagheb, K; Al-Nawas, B; Schiegnitz, E | CLINICAL ORAL INVESTIGATIONS | Excluded by abstract |
| 132 | Dental malpractice lawsuit cases in Saudi Arabia: A national study | Alsaeed, S; Aljarallah, S; Alarjani, A; Alghunaim, G; Alanizy, A | SAUDI DENTAL JOURNAL | Excluded by abstract |
| 133 | Alveolar bone loss and root resorption in mesialized second molars in mandibular first molar extraction cases as compared to contralateral non-extraction side in young adults: A retrospective cross-sectional study | Aman, M; Jeelani, W; Ahmed, M; Khalid, A | INTERNATIONAL ORTHODONTICS | Excluded by abstract |
| 134 | APSIC dental infection prevention and control (IPC) guidelines | Ling, ML; Ching, P; Cheng, J; Lang, L; Liberali, S; Poon, P; Shin, Y; Sim, C | ANTIMICROBIAL RESISTANCE AND INFECTION CONTROL | Excluded by abstract |
| 135 | A case of accidental aspiration of a dental cutter into the bronchopulmonary tree: clinical implications and legal considerations | Ovidio, Carnevale, Pantaleone | Minerva Stomatologica | Excluded by abstract |
| 136 | A Retrospective Analysis of Non-Sharps-Related Injuries in a Dental School | Jeon, Chong, Jin, Walsh, Zachar, Zafar | Int Dent J | Excluded by abstract |
| 137 | Adverse events during dental care for children: Implications for practitioner health and wellness | Nainar, Hashim | Ped Dentistry | Excluded by abstract |
| 138 | Adverse events in orthodontic practice: A review of the literature; [Eventi avversi in ortodonzia: Revisione della letteratura] | Vaquero-Nino, P.; Perea-Perez, B.; Labajo-Gonzalez, E.; Santiago-Saez, A. | DENTAL CADMOS | Excluded by abstract |
| 139 | Allergy and toxic reactions to local anesthetics | Malamed S | Dentistry Today | Excluded by abstract |
| 140 | Analysis of 415 adverse events in dental practice in Spain from 2000 to 2010 | Perea-Pérez B, Santiago-Sáez A, García-Marín F, Labajo-González E, Villa-Vigil A. | Med Oral Patol Oral Cir Bucal | Excluded by abstract |
| 141 | Blood and saliva contamination on protective eyewear during dental treatment | Bergmann, Lindorfer, Ommerborn | Cl Oral Investigations | Excluded by abstract |
| 142 | Dental radiographs: benefits and safety | No author | Journal of ADA | Excluded by abstract |
| 143 | Direct-to-consumer dental products advertising: The role of evidence-based methods | DeVizio | J Evid-Based Den Pr | Excluded by abstract |
| 144 | Early treatment with a slow maxillary niti spring-expander. Narrative review of the literature; [Espansore con molle a balestra in niti in early treatment. Revisione narrativa] | Brotto, Abate, Cavagnetto, Fama, Lucarelli, Esposito | DENTAL CADMOS | Excluded by abstract |
| 145 | Injuries to dental patients and visitors | Porter K, Porter S, Scully C, Theyer Y | J Dentistry | Excluded by abstract |
| 146 | Le Fort I maxillary osteotomy in a Jehovah’s Witness patient: strategies for minimizing blood loss and maximizing safety | O'Connor, Emanuelli, Garg | Max Pl and Rec Surg | Excluded by abstract |
| 147 | Little research on effective tools to improve patient safety in the dental setting | Bailey E, Tickle M, Campbell S, O'Malley L. | Evid Based Dent | Excluded by abstract |
| 148 | Optimizing quality and safety of dental materials | Dahl J, Stenhagen I | Eur J Oral Sc | Excluded by abstract |
| 149 | Orthodontic adhesive systems-over half a century of research and experience | Kusmierczyk D, Malkiewicz K | J Stomat | Excluded by abstract |
| 150 | Risks with dental materials | Tillberg A, Jarvholm B, Berglund A | Dental Materials | Excluded by abstract |
| 151 | Segmental Maxillary Osteotomies in Conjunction With Bimaxillary Orthognathic Surgery: Indications – Safety – Outcome | Posnick J, Adachie A, Choi E | J Oral Maxillofac Surg | Excluded by abstract |
| 152 | Side-effects of Dental Materials Reported in Scandinavian Countries | Bergmann Maud | Den Mat J | Excluded by abstract |
| 153 | The adaption and implementation of the WHO surgical safety checklist for dental procedures | Wright, Ucer, Crofts | BDJ | Excluded by abstract |
| 154 | The impact of wrong-site surgery on dental undergraduate teaching: a survey of UK dental schools | Dargue et al. | Eur J Den Edu | Excluded by abstract |
| 155 | Understanding Laser Safety in Dentistry | Dennis, Owens, Romanos | Adv Laser Sur in Den | Excluded by abstract |
