# Supplementary material for: Patient safety in orthodontic care: a scoping literature review with proposal for terminology and future research agenda
Source: BMC Oral Health. 2024 Jun 18;24:702. doi: 10.1186/s12903-024-04375-7 (PMC11184803; doi:10.1186/s12903-024-04375-7)
Supplement: Supplementary file 2 — Supplementary Material 2. [file 12903_2024_4375_MOESM2_ESM.docx]

Supplementary Table 2. Excluded studies after full text review with reasons for their exclusion. Also, added studies from reference lists.

| No | Title | Authors | Journal/Book | Status |
| --- | --- | --- | --- | --- |
| 1 | [X-ray examinations in orthodontic diagnostics as a source of ionizing radiation] | Rak D. | Bilt Udruz Ortodonata Jugosl | Full text unavailable/ contacted author |
| 2 | A historical review of the effects of dental radiography on pregnant patients | Flagler CK, Troici CM, Rathore SA. | J Am Dent Assoc | not assessing patient safety |
| 3 | Contemporary orthodontics: the micro-screw | Sharif MO, Waring DT. | Br Dent J | not assessing patient safety |
| 4 | Dental Infection Control | Upendran A, Gupta R, Geiger Z. | StatPearls | not relevant to orthodontics |
| 5 | Effectiveness and Safety of Minimally Invasive Orthodontic Tooth Movement Acceleration: A Systematic Review and Meta-analysis | Fu, T.; Liu, S.; Zhao, H.; Cao, M.; Zhang, R. | J Dent Research | not assessing patient safety |
| 6 | Eleven Basic Procedures/Practices for Dental Patient Safety | Perea-Pérez B, Labajo-González E, Acosta-Gío AE, Yamalik N. | J Patient Saf | not relevant to orthodontics |
| 7 | Eye safety in operative dentistry - a study in general dental practice | Farrier SL, Farrier JN, Gilmour AS. | Br Dent J | not relevant to orthodontics |
| 8 | Finding Dental Harm to Patients through Electronic Health Record-Based Triggers | Walji MF, Yansane A, Hebballi NB, Ibarra-Noriega AM, Kookal KK, Tungare S, Kent K, McPharlin R, Delattre V, Obadan-Udoh E, Tokede O, White J, Kalenderian E. | JDR Clin Trans Res | not relevant to orthodontics |
| 9 | Lessons learned from dental patient safety case reports | Obadan EM, Ramoni RB, Kalenderian E. | J Am Dent Assoc | not relevant to orthodontics |
| 10 | Optimizing quality and safety of dental materials | Dahl JE, Stenhagen ISR. | Eur J Oral Sci | not relevant to orthodontics |
| 11 | Patient safety in dental care: A challenging quality issue? An exploratory cohort study | Mettes T, Bruers J, van der Sanden W, Wensing M. | Acta Odontol Scand | not relevant to orthodontics |
| 12 | Patient safety in dental care: an integrative review | Corrêa CDTSO, Sousa P, Reis CT. | Cad Saude Publica | not relevant to orthodontics |
| 13 | Patient Safety Incidents in Primary Care Dentistry in England and Wales: A Mixed-Methods Study | Ensaldo-Carrasco E, Sheikh A, Cresswell K, Bedi R, Carson-Stevens A, Sheikh A. | J Patient Saf | not relevant to orthodontics |
| 14 | Piezocorticision-assisted orthodontics: Efficiency, safety, and long-term evaluation of the inflammatory process | Strippoli, Julien; Durand, Robert; Schmittbuhl, Matthieu; Rompre, Pierre; Voyer, Rene; Chandad, Fatiha; Nishio, Clarice | AJO-DO | not assessing patient safety |
| 15 | Preventing wrong tooth extraction in primary care oral surgery: developing local safety standards for invasive procedures (LocSSIPs) | Tagar HK. | Prim Dent J | not relevant to orthodontics |
| 16 | Intraoperative and Perioperative Complications in Anterior Maxillary Osteotomy: A Retrospective Evaluation of 103 Patients | Gunaseelan, Rajan; Anantanarayanan, Parameswaran; Veerabahu, Muthusubramanian; Vikraman, Baskarapandian; Sripal, Rajendran | J Oral and MaxFac Surgery | not assessing patient safety |
| 17 | Seeking adverse effects in systematic reviews of orthodontic interventions: protocol for a cross-sectional study | Steegmans PAJ, Bipat S, Meursinge Reynders RA. | Syst Rev | Study protocol |
| 18 | Temporomandibular disorders, trismus and malignancy: development of a checklist to improve patient safety | Beddis HP, Davies SJ, Budenberg A, Horner K, Pemberton MN. | Br Dent J | not relevant to orthodontics |
| 19 | Risk management strategies in orthodontics. Part 2: Administrative considerations | Abdelkarim and Jerrold | AJO-DO | not assessing patient safety |
| 20 | Does Orthodontic treatment harm children's diets? | Johal A, Al Jawad A, Marcenes W, Croft N | J of Dentistry | Added from reference list |
| 21 | Wrong tooth extraction: root cause analysis | Peleg, Givot, Halamish-Shani, Taicher | British Dental Journal | Added from reference list |
| 22 | The incidence and prevention of ocular injuries in orthodontic practice | A P Sims 1, T J Roberts-Harry, D P Roberts-Harry | Br J Orthod | Added from reference list |
